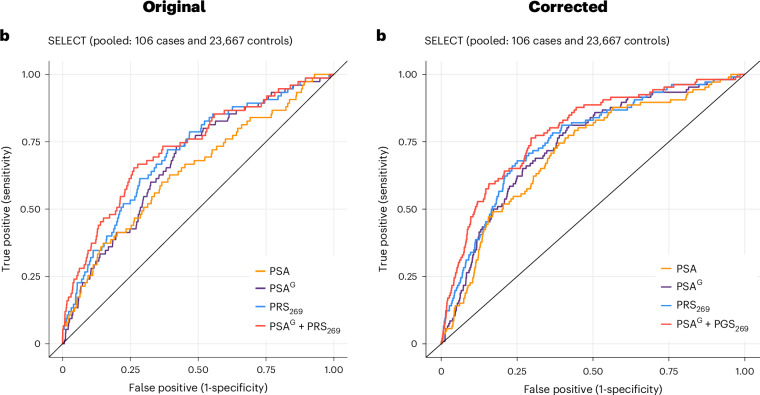# Author Correction: Genetically adjusted PSA levels for prostate cancer screening

**DOI:** 10.1038/s41591-025-03539-4

**Published:** 2025-02-03

**Authors:** Linda Kachuri, Thomas J. Hoffmann, Yu Jiang, Sonja I. Berndt, John P. Shelley, Kerry R. Schaffer, Mitchell J. Machiela, Neal D. Freedman, Wen-Yi Huang, Shengchao A. Li, Ryder Easterlin, Phyllis J. Goodman, Cathee Till, Ian Thompson, Hans Lilja, Stephen K. Van Den Eeden, Stephen J. Chanock, Christopher A. Haiman, David V. Conti, Robert J. Klein, Jonathan D. Mosley, Rebecca E. Graff, John S. Witte

**Affiliations:** 1 Department of Epidemiology & Biostatistics, University of California, San Francisco, San Francisco, CA USA; 2 Department of Epidemiology & Population Health, Stanford University School of Medicine, Stanford, CA USA; 3 Stanford Cancer Institute, Stanford University School of Medicine, Stanford, CA USA; 4 Institute of Human Genetics, University of California, San Francisco, San Francisco, CA USA; 5 Division of Cancer Epidemiology and Genetics, National Cancer Institute, Rockville, MD USA; 6 Department of Biomedical Informatics, Vanderbilt University Medical Center, Nashville, TN USA; 7 Vanderbilt-Ingram Cancer Center, Nashville, TN USA; 8 Biological and Medical Informatics, University of California, San Francisco, San Francisco, CA USA; 9 Fred Hutchinson Cancer Research Center, Seattle, WA USA; 10 SWOG Statistics and Data Management Center, Fred Hutchinson Cancer Research Center, Seattle, WA USA; 11 CHRISTUS Santa Rosa Medical Center Hospital, San Antonio, TX USA; 12 Departments of Laboratory Medicine, Surgery and Medicine, Memorial Sloan Kettering Cancer Center, New York, NY USA; 13 Department of Translational Medicine, Lund University, Skåne University Hospital, Malmö, Sweden; 14 Division of Research, Kaiser Permanente Northern California, Oakland, CA USA; 15 Center for Genetic Epidemiology, Department of Population and Preventive Health Sciences, Keck School of Medicine, University of Southern California, Los Angeles, CA USA; 16 Norris Comprehensive Cancer Center, Keck School of Medicine, University of Southern California, Los Angeles, CA USA; 17 Department of Genetics and Genomic Sciences, Icahn School of Medicine at Mount Sinai, New York, NY USA; 18 Department of Internal Medicine, Vanderbilt University Medical Center, Nashville, TN USA; 19 Departments of Biomedical Data Science and Genetics, Stanford University, Stanford, CA USA

**Keywords:** Prostate cancer, Cancer screening, Cancer genetics, Diagnostic markers

Correction to: *Nature Medicine* 10.1038/s41591-023-02277-9, published online 1 June 2023.

In the version of this article initially published, the graph shown in Fig. 6b was an inadvertent duplicate of Fig. 6a, while the key label now reading “PSA^G^ + PGS_269_” originally read “PSA^G^ + PRS_269_.” The figure has been amended in the HTML and PDF versions of the article.