# Supplementary material for: Genetically adjusted PSA levels for prostate cancer screening
Source: Nat Med. 2023 Jun 1;29(6):1412–23. doi: 10.1038/s41591-023-02277-9 (PMC10287565; doi:10.1038/s41591-023-02277-9)
Supplement: Supplementary file 2 — Reporting Summary [file 41591_2023_2277_MOESM2_ESM.pdf]

Corresponding author(s): Rebecca E. Graff, John S. Witte

Last updated by author(s): Feb 15, 2023

Reporting Summary

Nature Portfolio wishes to improve the reproducibility of the work that we publish. This form provides structure for consistency and transparency in reporting. For further information on Nature Portfolio policies, see our Editorial Policies and the Editorial Policy Checklist.

Statistics

For all statistical analyses, confirm that the following items are present in the figure legend, table legend, main text, or Methods section.

n/a	Confirmed	
☐	☒	The exact sample size (*n*) for each experimental group/condition, given as a discrete number and unit of measurement
☐	☒	A statement on whether measurements were taken from distinct samples or whether the same sample was measured repeatedly
☐	☒	The statistical test(s) used AND whether they are one- or two-sided<br>*Only common tests should be described solely by name; describe more complex techniques in the Methods section.*
☐	☒	A description of all covariates tested
☐	☒	A description of any assumptions or corrections, such as tests of normality and adjustment for multiple comparisons
☐	☒	A full description of the statistical parameters including central tendency (e.g. means) or other basic estimates (e.g. regression coefficient) AND variation (e.g. standard deviation) or associated estimates of uncertainty (e.g. confidence intervals)
☐	☒	For null hypothesis testing, the test statistic (e.g. *F*, *t*, *r*) with confidence intervals, effect sizes, degrees of freedom and *P* value noted<br>*Give P values as exact values whenever suitable.*
☒	☐	For Bayesian analysis, information on the choice of priors and Markov chain Monte Carlo settings
☒	☐	For hierarchical and complex designs, identification of the appropriate level for tests and full reporting of outcomes
☐	☒	Estimates of effect sizes (e.g. Cohen's *d*, Pearson's *r*), indicating how they were calculated

Our web collection on statistics for biologists contains articles on many of the points above.

Software and code

Policy information about availability of computer code

Data collection	No software was used for data collection.
Data analysis	- GWAS was performed using PLINK 2.0 (version 2.00a3LM)<br>- Fixed-effects inverse-variance-weighted meta-analysis was performed with METAL (version 2011), available from: http://csg.sph.umich.edu/abecasis/Metal/download/<br>- Heritability analyses were performed using LDAK (version 5.1), GCTA (version 1.93.2beta), and HDL (version 1.4.0), available from: https://github.com/zhenin/HDL<br>- Other statistical analyses and data visualizations were performed in R (version 4.1.2), including the use of the following R packages:<br>* SlopeHunter (version 0.0.2), available from: https://github.com/Osmahmoud/SlopeHunter<br>* Polygenic risk score modeling was performed using the PRS-CSx algorithm and reference panels, available from: https://github.com/getian107/PRScsx (version 1.0.0. July 29, 2021)<br>- Scripts for fitting polygenic scores, performing the index event bias analysis, and calculating genetically adjusted PSA values are available from: https://github.com/lkachuri/precision_PSA

For manuscripts utilizing custom algorithms or software that are central to the research but not yet described in published literature, software must be made available to editors and reviewers. We strongly encourage code deposition in a community repository (e.g. GitHub). See the Nature Portfolio guidelines for submitting code & software for further information.

Data

Policy information about availability of data

All manuscripts must include a data availability statement. This statement should provide the following information, where applicable:

- Accession codes, unique identifiers, or web links for publicly available datasets
- A description of any restrictions on data availability
- For clinical datasets or third party data, please ensure that the statement adheres to our policy

- The research was conducted with approved access to UK Biobank data under application number 14105 (PI: Witte). UK Biobank data are publicly available by request from https://www.ukbiobank.ac.uk.
- To maintain individuals' privacy, data on the GERA cohort are available by application to the Kaiser Permanente Research Bank (researchbank.kaiserpermanente.org).
- All PLCO genotype data is available in dbGaP18 under accession number phs001286.v2.p2 (https://identifiers.org/dbgap:phs001286.v2.p2). Companion phenotype data can be requested through the NCI Cancer Data Access System (CDAS) (https://cdas.cancer.gov/plco/). GWAS summary statistics are available directly from the PLCO Atlas GWAS Explorer website (https://exploregwas.cancer.gov/plco-atlas/) as well as accessed directly through API access (https://exploregwas.cancer.gov/plco-atlas/#/api-access).
- Scoring files for fitting PSA polygenic scores are available from the PGS Catalog: www.pgscatalog.org/score/PGS003378/ and www.pgscatalog.org/score/PGS003379/.
- Genome-wide summary statistics for the PSA multi-ancestry meta-analysis and ancestry-stratified summary statistics for the development of the genome-wide PSA polygenic score are available from: 10.5281/zenodo.7460135.

Human research participants

Policy information about studies involving human research participants and Sex and Gender in Research.

Reporting on sex and gender

Because PSA levels are only relevant to individuals with a prostate, all analyses in the study were restricted to individuals of male biological sex (as determined by analyses of genotype data using the Plink software). We additionally restricted to individuals with self-reported male gender identity in an effort to reduce variability in PSA levels attributable to discordance between biological sex and gender identity.

Population characteristics

- The UK Biobank is a population-based prospective cohort of 502,611 individuals from the United Kingdom, ages 40 to 69 at recruitment between 2006 and 2010. Age at PSA measurement ranged between 21.3 and 78.2 years (mean: 65.5 years). Median PSA across all available values was 2.35 ng/mL (mean: 4.24 ng/mL).
- The Resource for Genetic Epidemiology Research on Aging (GERA) Cohort consists of 100,000 adults who are members of the Kaiser Permanente Medical Care Plan, Northern California Region (KPNC), and participants in its Research Program on Genes, Environment and Health (RPGEH). This analysis used data from men aged between 20 and 90 years (mean: 64.9 years). Median PSA across all available values was 1.4 ng/mL (mean: 4.82 ng/mL)
- The Prostate, Lung, Colorectal and Ovarian (PLCO) Cancer Screening Trial is a completed randomized trial that enrolled approximately 155,000 participants aged 55 to 74 between November 1993 and July 2001. These analyses were limited to men (mean age: 62.5 years) with a baseline PSA measurement who were randomized to the screening arm of the trial (N=29,524) with mean PSA of 1.30 ng/mL (standard deviation = 0.88)
- In BioVU, the mean age at PSA measurement was 56.9 years and the median PSA level across all available measurements was 1.00 ng/mL.
- PCPT randomly assigned 18,880 men aged 55 years or older who had a normal DRE and PSA level ≤3 ng/mL to either finasteride or placebo. Analyses in this manuscript are based on 5883 participants aged between 55 and 85 years (mean: 63.2 years). Median PSA across all available values was 1.1 ng/mL (mean: 1.21 ng/mL)
- SELECT randomized 34,888 participants aged 50 or older between 2001 and 2004. The minimum enrollment age was 50 years for African American men and 55 years for all other men. Additional eligibility requirements included no prior prostate cancer diagnosis, ≤4 ng/mL of PSA in serum, and a digital rectal exam not suspicious for cancer. Analyses in this manuscript are based on 25,197 participants aged between 50 and 93 years (mean: 63.0 years). Median PSA across all available values was 1.1 ng/mL (mean: 1.30 ng/mL)
- Details of the participants included in the PSA GWAS from other studies have been previously described: Malmö Diet and Cancer Study (MDCS) [PMID: 22101116]

Recruitment

Since population-level PSA screening is not currently recommended, all observational PSA data is subject to some level of selection bias arising from patient and healthcare provider preferences, as well as variation in routine clinical care.

The UK Biobank is not representative of the general population across several sociodemographic, physical, lifestyle and health-related characteristics, with evidence of a "healthy volunteer" selection bias, details of which are published elsewhere (Fry et al, Am J Epidemiol 2017;186:1026-34. PMID 28641372). Analyses in the presented here are further restricted to a subset of men within the UK Biobank who had linked GP records with available PSA values.
GERA was developed from a mailed survey sent to all adult members of the Kaiser Permanente Medical Care Plan, Northern California Region (KPNC) who had been members for two years or more in 2007. The membership of KPNC is representative of the general population in the 14 county area in which facilities are located, although the membership is underrepresented for the extremes of income at both ends of the spectrum.
The Malmö Diet and Cancer Study (MDCS) is a population-based prospective cohort study that recruited men and women aged between 44 and 74 years old who were living in Malmö, Sweden between 1991 and 1996. These analyses included men from the MDCS who were not diagnosed with prostate cancer as of December 2014 and had available genotyping and baseline PSA measurement.
The Vanderbilt University Medical Center BioVU resource is a synthetic derivative biobank linked to deidentified electronic

health records. Analyses were based on PSA levels that were measured as part of routine clinical care.
PLCO enrolled approximately 155,000 participants aged 55 to 74 between November 1993 and July 2001. Men randomized to the screening arm of the trial underwent annual screening with PSA for six years and digital rectal exam (DRE) for four years. These analyses were limited to men randomized to the screening arm of the trial (N= 29,524).

PCPT randomly assigned 18,880 men aged 55 years or older who had a normal DRE and PSA level ≤3 ng/mL to either finasteride or placebo. Potential biases related to the PCPT design are discussed in detail by Goodman et al. (PMID: 16697846). In SELECT the minimum enrollment age was 50 years for African American men and 55 years for all other men. Additional eligibility requirements included no prior prostate cancer diagnosis, ≤4 ng/mL of PSA in serum, and a DRE not suspicious for cancer. Analyses presented in this manuscript included PCPT and SELECT participants who were genotyped on the Illumina Infinium Global Screening Array (GSAMD) 24v2-0 array.

Ethics oversight

Informed consent was obtained from all study participants. UK Biobank received ethics approval from the Research Ethics Committee (REC reference: 11/NW/0382) in accordance with the UK Biobank Ethics and Governance Framework. The Vanderbilt Institutional Review Board approved the BioVU study. We used previously published PSA GWAS results from the GERA cohort by Hoffmann et al. (PMID: 28139693). The original study was approved by the Kaiser Permanente Northern California Institutional Review Board and the University of California San Francisco Human Research Protection Program Committee on Human Research. The Malmö Diet and Cancer Study (MDCS) was approved by the local ethics committee. The PLCO study was approved by the institutional review board at each participating centre and the National Cancer Institute. The informed consent document signed by the PLCO study participants allows use of these data by investigators for discovery and hypothesis generation in the investigation of the genetic contributions to cancer and other adult diseases. Our study includes publicly posted genomic summary results from PLCO Atlas. No IRB review is required for PLCO summary data use.

Note that full information on the approval of the study protocol must also be provided in the manuscript.

Field-specific reporting

Please select the one below that is the best fit for your research. If you are not sure, read the appropriate sections before making your selection.

☒ Life sciences ☐ Behavioural & social sciences ☐ Ecological, evolutionary & environmental sciences

For a reference copy of the document with all sections, see nature.com/documents/nr-reporting-summary-flat.pdf

Life sciences study design

All studies must disclose on these points even when the disclosure is negative.

Sample size

Multi-ancestry genome-wide meta-analysis of PSA levels included a total of 95,768 men. This was the final sample size after all relevant exclusions (described below). Results of this analysis were used to develop a PSA genetic score that was validated in individuals that passed quality control in the Prostate Cancer Prevention Trial (n=5883) and the Selenium and Vitamin E Cancer Prevention Trial (n=22,173). These clinical trials have concluded and were converted to observational studies.

Data exclusions

Genome-wide association study (GWAS) of PSA levels excluded participants who were ever diagnosed with prostate cancer and individuals with PSA values >10 or PSA=0 (as an indicator of prostate resection). Analyses in the Prostate Lung, Colorectal and Ovarian (PLCO) were limited to men with a baseline PSA measurement who were randomized to the screening arm of the trial. In the UK Biobank we removed participants who withdrew consent at a later date and no longer wish their data to be included.
Additional exclusions focused on ensuring that only high-quality genetic data were retained for downstream analyses. Detailed descriptions of the quality control procedures performed by each contributing study are described in the Methods. Briefly, iterative 80% and 95% sample- and variant-level call rate filters were applied to remove poorly genotyped or contaminated samples and variants. Heterozygosity outliers within each ancestral population were detected using absolute values from PLINK method-of-moments F coefficients. Samples with values more than five standard deviations from the population mean were excluded. We also excluded individuals with discordant self-reported and genetically inferred sex based on X chromosome method-of-moments F coefficient from PLINK using 0.5 as the threshold (F coefficients are close to 0.0 for males and 1.0 for females). KING version 2.0 (http://people.virginia.edu/~wc9c/KING/)was used to estimate relatedness among the samples based on a subset of genotyped autosomal variants with minor allele frequency (MAF) ≥0.01 and genotype call rate ≥97%. We excluded one individual from each pair of first-degree relatives. To further minimize potential population stratification in the UK Biobank, we excluded individuals for whom either of the first two genetic ancestry principal components (PC's) were >5 standard deviations away from the mean of the population.
GWAS analyses were limited to variants with MAF>0.005 and imputation quality INFO>0.30 in each of the contributing studies. We excluded variants that were out of Hardy-Weinberg equilibrium in cancer-free individuals (p-value<1E-05 in UKB and p-value<1E-06 in some studies).

Replication

The goal of the present analysis is to establish the predictive performance the PSA genetic score and the potential clinical utility of using this score to correct PSA measurements. The PSA genetic score was developed from a GWAS of PSA levels in 95,768 men and was subsequently validated in two independent studies that were not part of the GWAS: the Prostate Cancer Prevention Trial (PCPT) and the Selenium and Vitamin E Cancer Prevention Trial (SELECT). We provide the genetic variants and corresponding weights (effect sizes) necessary to construct the PSA genetic score and perform genetic adjustment of PSA levels to facilitate future replication of this work.

Randomization

Observational studies (GERA cohort, Malmo Diet & Cancer Study) and biobanks (UK Biobank, BioVU) that contributed data to the PSA GWAS did not have a randomization or intervention component. Analyses in the PLCO were limited to baseline PSA values in the screening arm of the trial. GWAS of PSA levels adjusted for the following minimum set of covariates: age at PSA measurement, the first 10 genetic ancestry principal components, and genotyping array or imputation batch (where applicable). Association analyses of polygenic scores (PGS) and genetically adjusted PSA in relation to prostate cancer incidence that were performed in the Prostate Cancer Prevention Trial (PCPT) and the

Selenium and Vitamin E Cancer Prevention Trial (SELECT) included randomization arm as a covariate in addition to age and genetic ancestry principal components.

Blinding	All data for performing genome-wide association analyses and developing polygenic scores were de-identified.<br>The researchers who carried out the analyses for this manuscript had no influence on how the genotyping, PSA measurement, or assessment of cancer status was performed in any of the contributing studies.<br><br>Blinding is not relevant for our study because we used observational and EHR/biobank data (GERA, Malmo Diet & Cancer Study, UK Biobank, BioVU). The clinical trials that contributed data to our study have all been completed and converted to observational cohorts: Prostate, Lung, Colorectal and Ovarian (PLCO) Cancer Screening Trial, Prostate Cancer Prevention Trial (PCPT), and the Selenium and Vitamin E Cancer Prevention Trial (SELECT). For the purpose of our manuscript, blinding in these trials is not relevant since our analyses focused on baseline/pre-randomization PSA data for GWAS (in PLCO) and polygenic score validation (in PCPT and SELECT). For associations with prostate cancer in this manuscript, both PCPT and SELECT were analyzed as observational case-control studies.

Reporting for specific materials, systems and methods

We require information from authors about some types of materials, experimental systems and methods used in many studies. Here, indicate whether each material, system or method listed is relevant to your study. If you are not sure if a list item applies to your research, read the appropriate section before selecting a response.

Materials & experimental systems

n/a	Involved in the study
☒	☐ Antibodies
☒	☐ Eukaryotic cell lines
☒	☐ Palaeontology and archaeology
☒	☐ Animals and other organisms
☒	☐ Clinical data
☒	☐ Dual use research of concern

Methods

n/a	Involved in the study
☒	☐ ChIP-seq
☒	☐ Flow cytometry
☒	☐ MRI-based neuroimaging
